# Development and validation of the competence in evidence based practice questionnaire (EBP-COQ) among nursing students

**DOI:** 10.1186/1472-6920-13-19

**Published:** 2013-02-07

**Authors:** Maria Ruzafa-Martinez, Lidon Lopez-Iborra, Teresa Moreno-Casbas, Manuel Madrigal-Torres

**Affiliations:** 1 Nursing Department, Faculty of Nursing, University of Murcia, Edificio D. Campus de Espinardo, 30100, Murcia, Spain; 2 Nursing and Healthcare Research Unit (Investén-isciii). Instituto de Salud Carlos III. C/Monforte de Lemos, 5. Pabellón 13. Sótano 2, 28029, Madrid, Spain; 3 Universitary General Hospital Reina Sofia. General Surgical Unit, Avda. Intendente Jorge Palacios, 1, 30003, Murcia, Spain

**Keywords:** Competence, Evidence-based practice, Undergraduate nursing, Questionnaire, Validation

## Abstract

**Background:**

Nursing educators need rigorously developed instruments to assess competency in evidence based practice (EBP) at undergraduate level. This concept is defined as the capability to choose and use an integrated combination of knowledge, skills and attitudes with the intention to develop a task in a certain context. Also, we understand that EBP is gaining knowledge and skills, as well as increasing positive attitudes toward EBP that will promote a change in behaviour to implement EBP in practice. This study aims to develop a psychometric test of the Evidence Based Practice Evaluation Competence Questionnaire (EBP-COQ) among undergraduate nursing students.

**Methods:**

The questionnaire was developed by item generation through a review of scientific literature and focus groups. The instrument was validated in terms of content validity through an expert review. The EBP-COQ was administered to a cohort of nursing students (n =100) to evaluate test reliability and select the best items. Psychometric properties of the final instrument were assessed in a sample of 261 nursing students.

**Results:**

The EBP-COQ consisted of 25 items. A factorial analysis grouped the items into the three categories that define competence relating to EBP: attitude, knowledge and skills. Cronbach’s alpha was 0.888 for the entire questionnaire. The factor solution explained 55.55% of the variance.

**Conclusions:**

EBP-COQ appears to measure with adequate reliability the attributes of undergraduate nursing students’ competence in EBP. The instrument is quick to disseminate and easy to score, making it a suitable instrument for nursing educators to evaluate students’ self-perceived competence in EBP.

## Background

As a result of the Bologna Process the educational systems in all European countries are in the process of reforming and harmonization. Accordingly, at the European Higher Education Space the learning model has been built on competence-based education (CBE). The characteristics of CBE are: it is oriented to the professional practice; it is learner-centred and the learning process is central; it has a constructivist approach [1].

In Spain, from this new framework, the National Government has determined a revision of the curricula, the teaching model and a definition of the competences in most of the degrees, included health care sciences (nursing, medicine, etc.). In the new Nursing Degree (four-year programme) one of the academic competences is related to the utilization of “Evidence-based Practice (EBP)” in the clinical decision-making [2].

So, the nursing curriculum should provide the acquisition and development of knowledge, attitudes and skills in EBP. For successful implementation in practice, EBP knowledge would need to result in skills, attitudes and appropriate changes in behaviour [3]. Most evidence-based practice (EBP) educational assessment tools evaluated until today have focused on specific knowledge components or technical skills. Other important potential barriers to the adoption of EBP, such as attitudinal, perceptual and behavioural factors, have yet to be studied [4], especially in the undergraduate setting [5]. Shaneyfelt [6] added that further development and testing is required to evaluate EBP attitudes. Tilson et al. remark the significance of this concept “EBP is gaining knowledge and skills, as well as much as increasing positives attitudes toward EBP that will promote a change in behaviour to implement EBP in practice” [7]. Therefore, the teachers and health care educators need good quality instruments to assess the student acquisition of EBP competence.

Determination of the best methods to teach clinical decision-making has been made difficult by the lack of well-validated evaluation tools and the absence of randomized controlled trials evaluating the impact of EBP educational interventions [8]. In addition, assessment tools used to assess EBP competence have primarily focussed on medical students and graduates [9,10]. A systematic review [6] identified 104 unique instruments, most of which were administered to medical students and postgraduate trainees and evaluated EBP skills. That paper identified that the majority of instruments predominantly focused only on one aspect of EBP (critical appraisal).

At nursing area the published questionnaires are used to evaluate self perceived competency in EBP for registered nurses. One of this, the Evidence Based Practice Questionnaire (EBPQ) [11] focuses on research utilization, in particular nurses’ ability to access and appraise research reports and implement research findings in practice. Several other questionnaires have been adapted from medical context. One of them is the questionnaire adapted by Watters et al. [12] from the McColl et al. [13] Evidence Based Medicine instrument. Watters et al. didn’t mention the reliability and validity results from the questionnaire adaptation. Other example of the use of medicine instruments to study the EBP in nursing is the one written by Brown et al. [14] based in the Johnston et al. [5] “KAB Questionnaire”. In this case, it was focused in United Stated nursing students from different academic years, and the authors didn’t make any adaptation of the items to the nursing context.

Very few reports have looked at the undergraduate learning environment, even less in nursing context. A growing body of literature exploring EBP teaching and learning confirms the research deficit in undergraduate EBP education and calls for further work within this area. Waters et al. [12] adapted the Nurses Perceptions of Evidence- based Practice survey for student attending post-registration education courses at a professional nursing college. Brown et al. [14] adapted the questionnaire developed for undergraduate medicine students by Johnston et al. [5] to nursing students. To conclude: assessing EBP competence in nursing students is hampered by a relative shortage of validated and practical assessment tools.

In Spanish nursing area we only found two questionnaires related to the EBP. One is the translation of the EBPQ [15] and the other one has been originally developed in Spanish [16]. However, any of these have been created specifically for the assessment of undergraduate nursing student’s competence in EBP.

From this review of the literature and published instruments, there appeared to be a need for a knowledge, attitude and behaviour questionnaire designed to evaluate EBP teaching and learning in an undergraduate nursing curriculum in Spanish. However, concepts like attitude and behaviour need to be measure with self-perceived questionnaires. For the reason that undergraduate nursing students’ competence in EBP appear to be important in determining the future behaviour and use of EBP [7] and because existing instruments may be less than satisfactory for measuring these attributes. For the purpose of this study, competence is defined as the capability to choose and use an integrated combination of knowledge, skills and attitudes with the intention to develop a task in a certain context [17].

The purpose of the study was to develop and validate a new measure, the Evidence-based Practice Competence Questionnaire (EBP-COQ), an instrument to assess undergraduate nursing students’ attitude, knowledge and skills in EBP in a Spanish context.

## Methods

The development and validation of the EBP-COQ occurred in three phases. The first phase consisted of the identification of items for the questionnaire, the second phase involved a pilot test of the first version of the instrument, and the final phase comprised the processes used to psychometrically evaluate the questionnaire administering in our target population, undergraduate nursing students. The steps followed for the instrument’s development used guidelines and methodologies [18].

### Item development and selection

In phase one, a review of the literature was carried out by searching for papers and questionnaires related to the measurement of competence (attitude, knowledge and skills) in EBP. Scientific databases (PubMed, CINAHL, INDEX, EMBASE, Scielo and PsychInfo) were consulted looking for research published between 1990 and 2011. General descriptors were employed, “evidence” AND “based” AND “attitud*” OR”competenc*” AND “scale” OR “test” AND “measure*” OR “psychometr*” AND “student*” AND “teach*” AND “knowledge*” AND “skills*”. Educational and pedagogical publications were also reviewed. The search was limited to English and Spanish language. Types of papers selected were those describing instruments that measure nursing/medicine students, nurses’ or other health care workers’ competence about evidence-based practice. Reference lists of articles investigating this topic were also examined. The searching period began in September 2006 and ended in February 2012. Finally, 34 original questionnaires were reviewed. A thorough analysis was also performed to identify examples of items. Table 1 shows the most relevant articles reviewed for the items generation.

**Table 1:** Characteristic of questionnaire/scale reviewed for items identification

**Author**	**Questionnaire/scale**	**Items categories**	**Population**
Bennett KJSackett DL Haynes RB, Neufeld VR, Tugwell P & Roberts R (1987) [19]	- Critical Appraisal of the Clinical Literature to Medical Students	- Critical appraisal reading skills	Medical students
Landry FJ, Pangaro L, Kroenke K, Lucey C, Herbers, J. (1994) [20]	- Medical student knowledge of research design, basic critical appraisal skills, and attitudes toward and clinical use of the medical literature	- Critical appraisal reading knowledge	Medical students
		- Investigation design knowledge	
		- Skills in making clinical decision applying medical literature	
McColl A, Smith H & Field J (1998) [13]	General attitude toward Evidence-Based Medicine (EBM)	- Attitude towards EBM	General practitioners (UK)
		- Awareness and perceived usefulness of relevant extracting journals, review publications, and databases	
		- Ability to access relevant databases	
		- Understanding of technical terms used in EBM	
		- Views on the perceived major barriers to practicing EBM	
		- Views on how best to move from opinion based to EBM	
Fritsche L, Greenhalgh T, Falck-Ytter Y, Neumayer, HH, Kunz M. (2002) [21]	“Berlin Questionnaire”	-Knowledge about interpreting evidence	Medical students and residents
		-Skills to relate a clinical problem to a clinical question	
		-Best design to answer a question	
		-Use quantitative information from research to solve specific patient problems	
Ramos KD, Schafer S, Tracz SM. (2003) [22]	“Fresno test”	-Development of the investigation process: question formulation, adequate research design for this question, searching process and critical appraisal of the findings.	Medical residents
Johnston J M, et al (2003) [5]	Knowledge, attitude and behaviour questionnaire to evaluate Evidence-Based-Practice (EBP)	- Future use of EBP	Undergraduate medical school students (Hong Kong)
		- Attitudes towards EBP	
		- EBP knowledge	
		- Personal applications and use of EBP	
Aarons G A. (2004) [23]	Evidence-Based Practice Attitude Scale (EBPAS)	- Intuitive appeal of EBP	Mental Health service providers (USA)
		- Likelihood of adopting EBP	
		- Openness to new practices	
		- Perceived divergence of usual practice with research-based developed interventions	
Upton D & Upton P (2006) [11]	Evidence-Based Practice Questionaire	- Practice of EBP	Registered nurses different setting (Wales)
		- Attitude towards EBP	
		- Knowledge/skills associated with EBP	
Gerrish K, et al. (2007) [24]	Developing Evidence-Based Practice Questionnaire (DEBP)	- Bases of practice knowledge	Registered nurses (England)
		- Barriers to finding and reviewing evidence	
		- Barriers to changing practice on the basis of evidence	
		- Facilitation and support in changing practices	
		- Skills in finding and reviewing evidence	
Thiel L & Ghosh Y (2008) [25]	Nurses’ Readiness for EBP Survey	- Informational needs	Registered nurses (USA)
		- Evidence-based culture	
		- Perceived EBP knowledge	
		- Attitude toward EBP Scale (NATES)	
Melynk B M et al. (2008) [26]	EBP Beliefs Scale and EBP Implementation Scale	- Beliefs toward EBP	Registered nurses (USA)
		- EBP implementation	

For the second step in this phase, two focus groups were carried out in order to develop the items. This method allowed us to explore and identify relevant aspects of EBP for nursing students. We also extracted words and expressions that could be useful for refining and creating items on the questionnaire. The students were selected to maximize sample variation on criteria judged as likely to influence competence in EBP. The first group consisted of undergraduate nursing students who had studied EBP in their nursing programme as an optional course (n = 8) and the second consisted of nursing students who had not studied this matter (n = 8). A question guide was created to facilitate the discussion and to explore student opinions about their previous experience with EBP and their attitude toward its use and learning.

After the literature review and the focus groups a large pool of statements was prepared to be sure that there was an adequate sample of items within each of the three major content areas comprising the competence construct: attitude, knowledge and skills. The items were re-phrased into affirmative statements and asked the respondents to rate their self perception of competence in EBP using a 5-point Likert-type scale, ranging from 1 “Strongly disagree” to 5 “Strongly agree”. Each area of content was represented by equal numbers of positive and negative exemplars of the construct, a condition that tends to reduce subsequent error due to acquiescence. A pool of 110 potential items formed the first questionnaire version.

The selection of the items was carried out in two stages. In the first stage six experts in EBP were asked to classify the items into one of three dimensions that were part of the competence construct (attitude, knowledge and skills). The items which achieved an agreement level of less than 75% or raised doubts about their inclusion in one dimension or other were eliminated. For example, the item “I wish to be updated in the EBP implementation” was removed. A total of 28 items were eliminated, all of them from the attitude dimension. In a second stage the experts also evaluated the level of relevance of each item for its corresponding dimension of competence in EBP. The items were classified according to three categories: 3 “essential”, 2 “interesting but not essential” and 1 “irrelevant”. The statistical mean for each item was calculated and those, which had a mean over 2.5, were kept on the scale (relevance of 83.3%). The following version of the questionnaire was reduced to 62 items in a proportion that was the equivalent of items edited in a positive and negative sense.

In addition, 20 undergraduate nursing students were selected in order to assess the comprehension and feasibility of the reviewed pool of items and format response. They were selected with a socio-demographic and work profile that was similar to that of the study population.

The second phase and after modifying the items according the nursing students’ suggestions we administered the first draft of the EBP-COQ (62 items) to a convenience sample of second and third year nursing students enrolled at Faculty of Nursing in Spain. The day that the instrument was administered 148 students attended to class and 100 of them completed the questionnaire. The aims of this were to evaluate the quality of generated items and eliminate those proving to be inadequate.

### Description of the questionnaire

To collect more information and to use it for ulterior analyses we designed an instrument with 4 sections. The first section asked respondent to describe their personal and college characteristics, including age, gender, academic level, other degrees (Diploma, Bachelor, Master or PhD.), any extra training in EBP and nursing research carried out in the last 3 years, and number of journals that they had read in the last month. The next section was the EBP-COQ. A subscale of five items from the “Attitude to Research Scale” [27] was added as the third section. The last section explored the general attitude toward EBP, knowledge and skills in EBP, English language, statistic and computer practice measured on 10-point visual analogue scales. The objective of these 2 last sections was to facilitate the checking of the external construct validity of the questionnaire.

In phase three, the final version of the questionnaire was administered to undergraduate nursing students in November of 2009. The target population for this study was undergraduated nursing students from the Faculty of Nursing, University of Murcia. The convenience sample consisted of baccalaureate nursing students from second and third year (n = 325).

The study was approved by the Research Ethics Committee at University of Murcia. A participant information sheet giving details of the study accompanied the questionnaire. The consent to participate in the survey was personally asked to each student and confidence was assured.

Student participants were informed that participating or declining participation at the study would not affect grading, class standing, or further opportunities at the university.

### Data analysis

For the focus groups, all references to EBP competence were coded by two researches. Each researcher made a thematic analysis of the data, and coded them for quite specific concepts of EBP that has been extracted from the literature (believes, feelings, knowledge, implementation, usefulness, practice). A consensus was reached by checking the concepts and themes in relation to the coded extracts and the entire data set.

For the questionnaire, analyses were performed using SPSS version 17. Frequencies of all responses were reviewed for outliers and non-normality. Continuous variables were described using distribution, and ranges. *X*2 tests were used to compare competence in EBP at categorical variables (gender, previous training in EBP, etc). Student’s *t* test, analysis of variance testing, and correlation coefficients were used to compare competence in EBP and continuous variables (age, integration of research at work scale, visual analogue scales, etc…).

Cronbach’s alpha was used to quantify the internal reliability of the total questionnaire and the 3 factors, and to assess the contribution of each question to the overall reliability of each factor. Content validity was addressed through the instrument development process, both by basing the items on the prior instruments and by collegial development of the new items using a panel experienced in EBP.

Construct validity was carried out using pairwise deletion of missing values and conducting a principal components factor analysis with orthogonal varimax rotation for each item set. The sorted factor loadings, eigen values and scree plots resulting from these analyses were examined to identify the number of dimensions or factors that made up the best solution for each item set. We examined the factor loadings to determine whether all items in the set were associated with the attribute of interest. Items with a factor loading less than 0.40 were deleted.

External construct validity was also explored by examination of correlations between the attributes and other characteristics of the respondents as measured by selected other items of the survey. Specifically, we hypothesized that the competence in EBP should be highly intercorrelated with attitude to research, whereas the other attributes (previous training in EBP and Nursing Research) should be only modestly intercorrelated. In the special case of the visual analogue scale set addressing self-perception of attitude toward EBP, knowledge in English, biostatistic, etc were hypothesized to modestly intercorrelated with the EBP-COQ. Criterion-related validity was not addressed explicitly in this study.

## Results

Items development and selection has been explained in the Method section. In this section, we report the results of the pilot administration and the final validation of the EBP-COQ to the target population.

The sample population of undergraduate nursing students (n = 100) completed a pilot administration of the 62-item EBP-COQ. Cronbach’s alpha coefficient for the first administration was 0.881. Through an item analysis we discarded those items that were either highly correlated with other items, and were thus considered repetitive, or that had item-scale correlations less than 0.30. Eventually, with the reduction of these items a definitive scale was obtained to assess competence in EBP consisting of 32 items.

The final version of the EBP-COQ achieved an overall response rate of 80.30% (n = 261). The nursing students who participated in the survey were mainly women (82.5.5% [208] vs. 17.5% [44]). The sample characteristics are shown in Table 2. The minimum amount of data for factor analysis was satisfied, with a final sample size of 261 (using listwise deletion), with over 11 cases per variable. After an exploratory analysis of the lost data, it has been seen that in only four items there are seven lost cases, which is hardly more than 5% of non-responses for each item. Additionally, assuming that the items are treated as continuous variables, the descriptive statistical analysis was calculated, the floor and ceiling effect was tested, and the discriminatory capacity of the questions and their distribution were studied. In general all the responses of the items tend to be distributed among high values of the scale but without being grouped on one of the extremes, therefore, the distribution in the response categories is sufficiently wide.

**Table 2:** Socio-demographic and academy data of undergraduate nursing students

**Gender**	**N (%)**
Female	208 (82.5)
**Age**	**Mean (SD)**
	22.14 (5.305)
**Academic year**	**N (%)**
First year	4 (1.6)
Second year	172 (68.8)
Third year	74 (29.6)
**Other studies**	**N (%)**
None	207 (79.3)
Technical Diploma	37 (14.2)
Other degree (Diploma/Bachelor)	17 (6.5)
Master	______
PhD	______
**EBP training/education**	**N (%)**
None	191 (78.9)
< 40 hours	33 (13.6)
Between 40 and 150 hours	17 (7.0)
> 150 hours	1 (0.5)
**Research methodology training/education**	**N (%)**
None	156 (65.3)
< 40 hours	61 (25.5)
Between 40 and 150 hours	19 (7.9)
> 150 hours	3 (1.3)

N = number of cases.

SD = Standard Deviation.

### Validity analyses

As indicated previously, content validity for the item sets was addressed by grounding the questionnaire development in an earlier survey instruments, focus groups and in the development of the form with guidance from an expert panel.

### Instrument structure

During several steps, a total of seven items were eliminated because they did not contribute to a simple factor structure and failed to meet a minimum criteria of having a primary factor loading of 0.4 or above, and no cross-loading of 0.3 or above. For example, the item “I did not know the main healthcare bibliographic databases where I can search scientific information (Medline, Embase, Lilacs, CINALH, etc…)” did not load above 0.3 on any factor.

Finally, the factorability of the 25 items was examined. Several well-recognised criteria for the factorability of a correlation were used. Firstly, the 25 items correlated at least 0.3 with at least one other item, suggesting reasonable factorability. Secondly, the Kaiser–Meyer–Olkin measure of sampling adequacy was 0.933 (p < 0.001), suggesting that factor analysis was appropriate for this data set. Barlett’s test of sphericity was significant (χ^2^ (300) = 3037.995, *p* < 0.001). The diagonals of the anti-image correlation matrix were all over 0.5, supporting the inclusion of each item in the factor analysis. Finally, the communalities were all above 0.3 further confirming that each item shared some common variance with other items. Given these overall indicators, factor analysis was conducted with all 25 items.

The exploratory factor analysis (principal components) of the remaining 25 items, using varimax rotation to account for the relationship among the factors, yielded a three-factor structure that explained 55.55% of the variance of the data. Factor 1 (13 items): “Attitude toward EBP” consisted of items A2, A3, A4, A5, A6, A8, A9, A10, A11, A12, A14, A15 and A16 explained 33,46% of the total variance (eigenvalue 8.36); factor 2 (6 items): “Skills in EBP” consisted of items C1, C2, C4, C5, C6 and C7 explained 17,07% of the variance (eigenvalue 4.27); and factor 3 (6 items): “Knowledge in EBP” consisted of items C8, CQ0, C11, C12, C13 and C14 explained 5,03% of the total variance (eigenvalue 1.26). Table 3 presents the items with their loadings in each factor.

**Table 3:** Factor loadings and communalities for 25 items from the Evidence-based Practice Competence Questionnaire (EBP-COQ) (N = 261)

	**Attitude toward EBP**	**Skills in EBP**	**Knowledge in EBP**	**Communality**
A2 The EBP helps to make decisions in clinical practice	,769			,615
A3 I’m confident that I will be able to evaluate critically the quality of a scientific article	,603			,481
A4 The practice of EBP will help to have a better definition of the nurse roll	,822			,680
A5 The nursing contract should include time to read scientific papers and make critical appraisal of them.	,679			,466
A6 The widespread EBP implementation will allow to increase nursing autonomy from others professions.	,816			,670
A8 When I work as a nurse I will pleased if the PBE will be in practice	,789			,647
A9 The application of EBP improves patient’s healthcare outcomes	,847			,733
A10 In the future I wish to contribute to apply the EBP	,777			,606
A11 I do not like reading scientific articles	,668			,504
A12 The patient care will experiment minor changes with the EBP application	,762			,587
A14 It pleased me that the EBP is only a theoretical movement that does not takes in practice	,764			,609
A15 If I will have the opportunity I would assist to an EBP course	,640			,412
A16 I would like to have better access to published nursing scientific evidences	,724			,541
C1 I feel able to make a clinical question to start the searching of the best scientific evidence.			,665	,473
C2 I do not feel able to search for scientific evidences in the principles heath sciences data bases.	,423		,536	,469
C4 I do not feel able to search for the scientific information about the subject in the most important bibliographic indexes.	,429		,572	,512
C5 I feel able to evaluate critically the quality of a scientific article.			,632	,551
C6 I do not feel able to analyze if the obtained results of a scientific study are valid.		,402	,532	,447
C7 I feel able to analyze the practical utility of a scientific study.			,699	,571
C8 I know how to make clinical questions organize in the PICO format.		,587		,540
C10 I know the principal sources that offer the information revised and catalogued behind the evidence point of view.		,587		,521
C11 I do not know the most important characteristics of the principal investigation designs.		,755		,632
C12 I know the different evidence level of the designs of the investigation studies.		,784		,648
C13 I do not know the different recommendation grades about the adoption of a determined procedure or health intervention.		,670		,474
C14 I know the principal measures of association and potential impact that allow to evaluate the magnitude of the analyzed effect in investigation studies		,603		,498
Factor loadings < 0.4 are suppressed				

### Reliability

Cronbach’s alpha was 0.888 for the entire questionnaire. Internal reliability was also confirmed for each of the subscales with Cronbach’s alpha being 0.940 for factor 1: Attitude toward EBP; 0.756 for factor 2: Skills in EBP and 0.800 for factor 3: Knowledge in EBP.

External construct validity was also established by exploring the correlation between questionnaire scores and other variables that have been supposed are related to the competence in EBP concept. Tables 4 and 5 report correlation coefficients among the attribute scores and specific other items in the questionnaire that were employed in the construct validation analyze. A positive and high relationship was found between “attitude toward research” and EBP competence (global score) and factor 1: “attitude toward EBP”. However, there is not relationship with factor 3: “knowledge in EBP” and the correlation with the factor 2: “skills in EBP” is moderate (Table 4).

**Table 4:** Correlations among EBP-COQ dimensions and attitude toward research

**Attitude toward research**	**Attitude toward EBP**	**Skills in EBP**	**Knowldege in EBP**	**Global score (Competente in EBP)**
Pearson correlation	,750(**)	,252(**)	-,071	,618(**)
P valor	,000	,000	,276	,000
N	229	247	239	217

** p < 0,01 (bilateral).

**Table 5:** Correlations between EBP-COQ dimensions and the eight visual analogue scales

	**Attitude toward EBP**			**Knowledge in PBE**			**Skills in PBE**		
	**Pearson correlation**	**P value**	**N**	**Pearson correlation**	**P value**	**N**	**Pearson correlation**	**P value**	**N**
Self reported attitude toward EBP	,183^**^	,006	228	,250^**^	,000	241	,261^**^	,000	249
Skill level in EBP	,078	,242	228	,469^**^	,000	241	,349^**^	,000	248
Knowledge level in EBP	,102	,128	226	,494^**^	,000	239	,339^**^	,000	247
Attitude toward promotion	,102	,127	226	,235^**^	,000	240	,200^**^	,002	246
Perceived mates attitude toward EBP	-,091	,178	223	,262^**^	,000	236	,159^*^	,013	241
Knowledge in English Language	-,014	,838	227	,068	,294	240	,128^*^	,044	248
Knowledge level in computer	,037	,574	228	,241^**^	,000	241	,259^**^	,000	249
Knowledge level in statistics	-,004	,955	228	,104	,109	241	,117	,066	249

** p < 0.001 (bilateral); *p <0.05 (bilateral).

A sizable and significant positive correlation is present between factor 2 and 3 and the perception of knowledge level and skills level measured through a visual analogue scales (Table 5). Other correlations are smaller, even though some are significant. However, we should notice that factor 1 only correlates with self reported attitude toward EBP scale.

Discriminant validity was assessed by comparing those nursing students with previous training in EBP and research methodology and those without. The results of the Student’s *t*-test used to compare independent means indicated that those who have receive formal education in EBP and research methodology had a better self perception of Knowledge and Skills in EBP. The attitude toward EBP is also higher at those nursing students with training in EBP and Research although the different are only near significant (Table 6).

**Table 6:** Relation between attitude toward EBP, knowledge and skills in EPB and training in EBP/Research Methodology

	**Training in EBP**	**N**	**Score**	**Standard desviation**	**P value**
Attitude toward EBP	NO	172	3,66	,677	0.125
	YES	46	3,84	,813	
Knowledge in EBP	NO	180	2,65	,723	0.000
	YES	50	3,23	,636	
Skills in EBP	NO	185	3,19	,639	0.006
	YES	51	3,46	,528	
	**Training in Research Methodology**	N	Score	Standard Desviation	P value
Attitude toward EBP	NO	138	3,63	,722	0.076
	YES	77	3,81	,683	
Knowledge in EBP	NO	146	2,60	,725	0.000
	YES	81	3,07	,696	
Skills in EBP	NO	151	3,12	,614	0.000
	YES	82	3,47	,576	

## Discussion

This paper described the development and psychometric evaluation of a questionnaire designed to explore nursing undergraduate students’ self-perceived competence in EBP. It consists of 25 items, which are organized into three subscales (attitudes towards EBP, skills and knowledge of EBP). All items of the instrument are scored on a Likert-type scale of 1- 5, with a higher score indicating more self-perceived competence in EBP, greater self-perception of knowledge and skills in EBP, and more positive attitudes towards the EBP. Cronbach’s alpha measured 0.88 for the entire questionnaire, demonstrating internal consistency.

Although the instrument is focused in nursing undergraduate students, the selection of the content included in the questionnaire was based on both, relevant literature, professional experts and nursing students, in order not to omit most important issues. These procedures, in addition with theoretical definition of the constructed covered by the instrument, and with experts review over items, contributed to support the face validity [28]. Construct validity was established through the demonstration of convergent and discriminant validity.

### Limitations

Criterion validity has not been measured as an agreement in terms of responses collected from a gold standard questionnaire. At the moment that the study was carried out, any Spanish gold standard was known. However, discriminant validity was aimed at examining the ability of the EBP-COQ to distinguish between groups so that it should theoretically be able to distinguish between those nursing students with previous knowledge of EBN and those without. On the other hand, the correlation between attitude towards research and the self perception of knowledge, skills and attitude measured through the analogue visual scales and EBP-COQ scores suggested that the questionnaire was measuring competency in EBP.

### Implications

As similar publications aren’t available at this point, this work posts a contribution to the educational research. The Sicily Consensus Statement on EBP [7,29] high- lighted a need for effective training in each of the five steps of EBP, and future research into valid and reliable instruments to evaluate this training. The present questionnaire could be a useful measure to evaluate the programmatic impact of EBP educational interventions in nursing area. Educators might turn to instruments with strong evidence of responsive validity in order to recommend appropriate educational and organizational interventions. This could contribute to reduce the lack of consensus as to the best teaching and learning methods for integrating EBP into an undergraduate-nursing curriculum.

The choice of an EBP evaluation instrument should be guided by the purpose of the evaluation and the EBP domains of interest. Future users of the questionnaire should be into account that the definition of the EBP competence by the Tuning Nursing Project [30] and the technical statements from the Nursing Curricula has oriented the scope of its content.

As previously mentioned other authors have developed questionnaires with the aim to evaluate EBP in health related professions [5,11,31]. One example of it is the KAB questionnaire, one of the most referenced instruments in this context. Johnston et al. [5] designed the “KAB” questionnaire with a result of a 50, 7% of the variance. The Cronbach’s alpha of each of the factors of Johnston et al questionnaire were between 0,75 to 0,88. If we compare these statistical results with the EBP-COQ ones, we can highlight the relevance of the EBP-COQ instrument.

A crucial characteristic of the EBP-COQ is the self-perceived method of assessment. The intention was to use an approach that allows assessing the subject’s attitude, one of the three components of the competence construct. This is one of the most important differences from other traditional instruments that hardly assess this domain in depth because of the difficulty to assess behavioural issues through objective measures. However, previous studies have been able to demonstrate the importance of this dimension as a predictor of a positive behavioral toward resource utilization after following educational interventions [7,32].

On the other hand, the use of objective or self-perceived instruments to measure knowledge and skills depends on the research aims and both of them could be complementary. McCluskey and Lovarini [33] have showed that objective and self-perceived instruments found similar results in a pre-post test educational intervention addressing in a sample of occupational therapists.

In addition, the self-perceived method is the most useful to way to measure the competence in EBP even before the students receive the training, or it could be used with the same group of participants (e.g. pre and post EBP training). This solves one of the issues concerning development of EBP evaluation instruments whether the instrument is intended for repeated use [34]. As McCluskey and Lovarini [33] suggest a limitation of the objective measure instruments is the possibility of a learning effect from repeated administration of the outcome measures. Repeated administration of measures, particularly those focusing on knowledge, may have over-estimated the treatment effect.

## Conclusions

The EBP-COQ instrument assesses the self-perceived competence level in EBP. The instrument has demonstrated very good reliability, and the validity findings show promise in the application of the instrument for evaluating change due to education at an undergraduate nursing level.

In contrast to other EBP assessment instruments that focus primarily on comprehension of concepts, the EBP-COQ provides information about the effects of training in three domains: attitudes, knowledge and skills. Overall, the EBP-COQ demonstrates good sensitivity to the effects of training, distinguishes among respondents with different educational training in EBP and research methodology, has good reliability and has strong internal consistency when the instrument is considered as a whole, across three dimensions.

The self-report and multiple-choice design ensured the use of the instrument as reflected in the short completion times. The instrument is applicable to classroom settings, workshops, seminars, faculty retreats, and online administration.

Future steps of this research could be focused on a confirmatory factor analysis of the preliminary questionnaire version presented here, in other Spanish representative sample, or even in other Spanish language countries. These researches could explore additional evidences of validity based in construct, convergent and criterion indicators.

## Competing interest

The authors declare that they have no competing interests.

## Authors’ information

MARÍA RUZAFA-MARTÍNEZ RN MSc PhD is a lecturer of the University of Murcia Faculty of Nursing. She teaches EBP in undergraduate education. Her investigation interest is focused in development and validation of questionnaires and implementation of EBP.

LIDÓN LÓPEZ-IBORRA RN PhD is an associated professor of the University of Murcia Faculty of Nursing. She teaches EBP in undergraduate education. Her investigation interest is focused in development and validation of questionnaires and implementation of EBP.

TERESA MORENO-CASBAS RN MSc PhD FEAN is the Director of the Nursing and Health Care Research Unit from the Institute of Health Carlos III. Her investigation interest is focused in the implementation of Evidence Based Practice in clinical settings.

MANUEL MADRIGAL-TORRES MSc PhD is associated professor of the University of Murcia Faculty of Medicine. His investigation interest is focused in development and validation of questionnaires.

## Authors’ contributions

MRM, LLI, TMC and MMT all originated and conceived of the study, and participated in the drafting and editing of the manuscript. All authors read and approved the final version of the manuscript. All authors contributed equally to this work.

## Pre-publication history

The pre-publication history for this paper can be accessed here:

http://www.biomedcentral.com/1472-6920/13/19/prepub
